# ﻿Taxonomic review of *Kaloplocamus* from the Yellow Sea, China with the description of a new species (Nudibranchia, Doridina, Polyceridae)

**DOI:** 10.3897/zookeys.1168.101248

**Published:** 2023-06-27

**Authors:** Jingcheng Wei, Lingfeng Kong

**Affiliations:** 1 Key Laboratory of Mariculture, Ministry of Education, Ocean University of China, Qingdao, 266003, China; 2 Sanya Oceanographic Institution, Ocean University of China, Sanya 572000, China; 3 Laboratory for Marine Fisheries Science and Food Production Processes, Laoshan Laboratory, Qingdao 266237, China

**Keywords:** Biodiversity, phylogenetics, pseudocryptic species, Yellow Sea

## Abstract

Species of *Kaloplocamus* Bergh, 1880 are enigmatic Nudibranchia sea slugs, and only two valid species are reported in the northwestern Pacific. *Kaloplocamusjaponicus* (Bergh, 1880) was initially described based on alcohol-fixed specimens. In the latest revision of *Kaloplocamus*, it was synonymized with *Kaloplocamusramosus* (Cantraine, 1835). Recently, several nudibranchs were collected from Tianheng, Shandong Province, China, and one of them is identified as an undescribed species described here as *Kaloplocamusalbopunctatus***sp. nov.** based on integrated approaches incorporating morphological observations, internal anatomy, and phylogenetic analyses of two mitochondrial (COI, 16S rRNA) genes. The other species is identified as *K.japonicus* Bergh, 1880 based on the anatomy of the reproductive system. The new species *K.albopunctatus***sp. nov.** is similar to *K.ramosus* in having a bright orange-red color pattern but differs significantly in the structure of appendages and reproductive system. *Kaloplocamusjaponicus* can be easily distinguished from other *Kaloplocamus* species by its translucent, white-pink coloration and unique features of the female reproductive organ. Both species are supported as distinct species in all molecular analyses. The phylogenetic analyses propose a new estimate of the relationship between *Kaloplocamus* and *Plocamopherus*, and the evolution of bioluminescence within Triophinae is discussed. Our results also suggest cryptic biodiversity within the *K.ramosus* species complex.

## ﻿Introduction

Triophinae Odhner, 1941 includes a group of sea slugs with elongated bodies and ramified appendages along the margin of the reduced mantles. Among them are some species that are capable of bioluminescence, which is only reported within few genera of Nudibranchia including *Kaloplocamus* Bergh, 1880 and *Plocamopherus* Rüppell & Leuckart, 1828 (Wilbur and Yonge 1964). *Kaloplocamus* Bergh, 1880 is characterized by having appendages without a globular structure on the tip and a reproductive system lacking a penial sac. The genus was initially known as *Euplocamus* Philippi, 1836, but that name is a junior homonym (Vallès and Gosliner 2006) and the valid name is *Kaloplocamus*, described by Bergh (1880) and among several suggested available names for it. Few revisions of this genus have been made, and it was not until early in the 21^st^ century that the first thorough revision of *Kaloplocamus* was published (Vallès and Gosliner 2006), with three new species described. Baba (1989) described three forms (orange form A, orange-spotted form B, and dark-mottled form C) of *K.ramosus* (Cantraine, 1835), as well as *K.acutus* Baba, 1955 from Japan. Orange form A was identified as *K.ramosus* based on the descriptions of Bergh (1880) and Vayssière (1901), and orange-spotted form B and dark-mottled form C were considered to *K.ramosus* based on the number of appendages and the radular morphology. As *K.ramosus* was redescribed by Vallès and Gosliner (2006), the identification of Baba’s specimens should also be revised.

To date, there are six valid species in this genus, but few of them are well studied, despite the fact that knowing more about these bioluminescent animals may throw light on the origins and evolution of bioluminescence within the nudibranchs. Moreover, Vallès and Gosliner (2006) did not include a phylogenetic analysis in their work. Recent phylogenetic studies have not included sufficient species and the relationships of taxa within Triophinae are still controversial (Palomar et al. 2014; Hallas et al. 2017; Korshunova et al. 2020).

Bergh (1880) described *Euplocamusjaponicus* Bergh, 1880 based on three alcohol-fixed specimens from Japan. Later, Eliot (1913) reported two alcohol-fixed specimens from Moroiso, Japan, and doubted if these specimens were *E.japonicus* as they shared many similarities with *Euplocamuscroceus* R.A. Philippi, 1836. In the latest revision of *Kaloplocamus* (Vallès and Gosliner 2006), *K.japonicus* was synonymized with *K.ramosus* following the remarks of Eliot (1913). However, Eliot (1913) only reported several external characters, the radula, and the structure of the vas deferens, while Bergh (1880) described the external morphology and internal anatomy of his specimens in detail. Therefore, the validity of *K.japonicus* remains to be discussed.

Vallès and Gosliner (2006) synonymized *K.yatesi* (Angas, 1864), *K.orientalis* Thiele, 1925, *K.aureus* Odhner, 1932, and *K.filosus* Cattaneo-Vietti & Sordi, 1988 with *K.ramosus* due to their poor descriptions and unclearly defined characters, making *K.ramosus* a widespread species whose geographic distribution comprised of the Mediterranean Sea, Atlantic Ocean, and western Pacific including the Yellow Sea of China (Qi et al. 1986, 1989; Zhang et al. 2016). However, in recent years, more and more formerly thought widespread species have turned out to be a complex of morphologically similar species (Krug et al. 2013; Chichvarkhin et al. 2016; Mehrotra et al. 2020). These species, usually referred to as “pseudocryptic species”, are similar in appearance, which makes it difficult to distinguish them by external characters, contributing to misidentification and confusion in the use of species names. To unmask the potential biodiversity of pseudocryptic species proposes new challenges for taxonomic workers. Newly developed molecular technologies, such as ABGD (Automatic Barcode Gap Discovery; Puillandre et al. 2012) and ASAP (Assemble Species by Automatic Partitioning; Puillandre et al. 2021) methods, might provide new insights to species delimitation and have been applied to solving several pseudocryptic species problems (Tibiriçá et al. 2018; Ekimova et al. 2020).

To investigate the phylogeny of Triophinae and pseudocryptic species problems within *K.ramosus*, we examined the external morphology, anatomy of the reproductive system, characters of radulae, and egg masses of two species collected from Tianheng, Shandong Province, China. Phylogenetic analyses based on mitochondrial cytochrome c oxidase subunit I (COI) and 16S rRNA (16S) genes were conducted to decipher the relationships of these species and their congeners. Based on integrated morphological and molecular analyses, *Kaloplocamusalbopunctatus* sp. nov. is described. *Kaloplocamusjaponicus* is revised based on the anatomy of reproductive system. Additionally, the species complex surrounding *K.ramosus* is discussed using the ASAP species delimitation analysis.

## ﻿Materials and methods

### ﻿Taxon sampling and morphological studies

**Figure 1.**
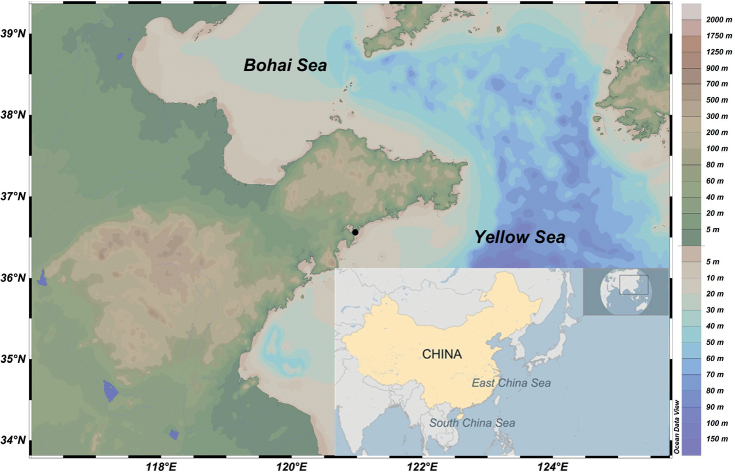
Map of sampling site (black dot) of *Kaloplocamusalbopunctatus* sp. nov. and *K.japonicus* (36°33.7′N, 120°58.6′E). Map courtesy of Ocean Data View 5.6.3 (Schlitzer 2021; http://odv.awi.de).

Specimens were collected from the intertidal zone of the seashore in Tianheng, Shandong Province, China (Fig. 1), then brought to the laboratory and maintained in a seawater tank. Living animals and egg masses were observed and photographed with a Nikon D4 camera (Nikon, Tokyo, Japan). Afterwards, the specimens were fixed in 95% ethanol for molecular and anatomical work. Fixed specimens were dissected under a stereomicroscope (Nikon SMZ 800N; Nikon, Tokyo, Japan), the reproductive systems were photographed with the aid of a DS-Fi2 digital camera (Nikon, Tokyo, Japan) mounted on the stereomicroscope. Buccal masses were also obtained from fixed specimens and were placed in 10% NaOH for 24 h until the muscle tissue was completely dissolved, from which radula was removed and rinsed in double-distilled water before being gilded and examined under a Tescan Vega3 scanning electron microscope (Tescan, Brno, Czech Republic). For the new species, type specimens were selected and deposited in the Laboratory of Shellfish Genetics and Breeding (**LSGB**) and Marine Biological Museum of Chinese Academy of Sciences (**MBMCAS**). The sampling locations were mapped using Ocean Data View 5.6.3 (Schlitzer 2021).

### ﻿DNA extraction, amplification, and sequencing

Genomic DNA was extracted from small pieces of foot of fixed specimens using the TIANamp Marine Animals DNA Kit (Tiangen, Beijing, China). Partial sequences of COI gene were amplified using the primer sequences from Folmer et al. (1994), with the forward primer LCO1490 (5ʹ-GGTCAACAAATCATAAAGATATTGG-3ʹ) and reverse primer HCO2198 (5ʹ-TAAACTTCAGGGTGACCAAAAAATCA-3ʹ). Primer sequences from Criscione and Ponder (2013) were used for the amplification of partial 16SrRNA gene sequences, with the forward primer 16SARis (5ʹ-TGCCTGTTTAGCAAAAACAT-3ʹ) and reverse primer 16SBRis (5ʹ-CCGGTCTGAACTCAGATCATGT-3ʹ). Polymerase chain reactions (PCRs) for two fragments were conducted in a reaction volume of 25 μl including 22 μl of Golden Mix v. 2 (Dye Plus; Tsingke, Beijing, China), 1 μl of both primers and 1 μl of DNA template. Thermal cycling for COI gene was performed as followed: an initial denaturing step at 94 °C for 3 min, 36 cycles of 30 s at 94 °C, 30 s at 48 °C (annealing temperature), 1 min at 72 °C for extension, and a final extension of 10 min at 72 °C. Thermal cycling for 16S rRNA gene was: an initial denaturing step at 94 °C for 5 min, 40 cycles of 30 s at 94 °C, 30 s at 52 °C (annealing temperature), 1 min at 72°C for extension, with a final extension of 10 min at 72 °C. The products were verified using 1.5% (w/v) agarose gel electrophoresis and checked under a UV transilluminator (Peiqing, Shanghai, China). Successfully amplified products were sequenced by BGI Co., Ltd (Shenzgen, China).

In the case of *Kaloplocamusjaponicus*, the COI gene could not be amplified by the universal primers. Consequently, the DNA template was sent to Novogene Technology Co., Ltd (Beijing, China) for library construction and high-throughput sequencing. Sequencing libraries with average insert sizes of 300 bp were built and then sequenced as 150 bp paired-end runs on the Illumina NovaSeq 6000 platform. Spades v. 3.12.0 was used for de novo mitochondrion assemblies (Bankevich et al. 2012). Complete mitochondrial genomes were obtained by BLASTN against the complete mitochondrial genome of *Polycerahedgpethi* (NCBI MZ713367.1); partial sequences of COI gene were then obtained by BLASTN against the sequence of *K.albopunctatus* sp. nov. and modified by hand.

### ﻿Phylogenetic analyses

COI and 16S sequences of Triophinae were downloaded from GenBank (Table 1). Sequence alignment for both genes was independently done by MAFFT v. 7.480 (Katoh and Standley 2013). Obscure sites for 16S sequence alignment were removed using Gblocks v. 0.91b with default parameters (Talavera and Castresana 2007). Trimmed sequences were concatenated using FASconCAT v. 1.0 (Kück and Meusemann 2010) as the dataset for subsequent analyses. Phylogenetic analyses were carried out using maximum-likelihood (ML) and Bayesian inference (BI) methods. IQ-TREE v. 2.4.1 was used for ML analysis with node support assessed by 1000 bootstrap replicates (Nguyen et al. 2015), the --modelomatic parameter was applied to search for the best-fit model and the GTR+F+I+G4 model was chosen according to the Bayesian Information Criterion (Whelan et al. 2015). As for the BI analysis, the best partition scheme was selected with PartitionFinder v. 2.1.1 (Lanfear et al. 2017), on which was based the BI analysis conducted via MrBayes v. 3.1.2 (Ronquist et al. 2012) on the CIPRES Science Gateway v. 3.3 (Miller et al. 2010), with four simultaneous Monte Carlo Markov chains (MCMC) running for 10 million generations. Trees were sampled every 1000 generations and the first 25% generations were discarded as burn-in. The *p*-distance based on the COI gene of *Kaloplocamus* species was calculated using MEGAX (Kumar et al. 2018). An Assemble Species by Automatic Partitioning (ASAP) analysis with default parameters was performed for the species delimitation of *K.ramosus* species complex (Puillandre et al. 2021).

**Table 1.** Sequences used in this study with collection details and accession numbers (new sequences are in bold).

Species	Location	COI	16S	Source
*Kalingaornata*	Vietnam	MN224072	MN224103	Korshunova et al. 2020
***Kaloplocamusalbopunctatus* sp. nov.**	**Tianheng, China**	**OP903111**	**OP908059**	**This study**
	**Tianheng, China**	**OP903112**	**OP908060**	**This study**
	**Tianheng, China**	**OP903113**	**OP908061**	**This study**
*Kaloplocamusramosus*	Australia	JX274104	JX274066	Palomar et al. 2014
	Portugal	EF142904	–	Pola et al. 2007
***Kaloplocamusjaponicus***	**Tianheng, China**	**OP903108**	**OP908062**	**This study**
	**Tianheng, China**	**OP903109**	**OP908063**	**This study**
	**Tianheng, China**	**OP903110**	**OP908064**	**This study**
*Kaloplocamus* sp. 1	Madagascar	MF958429	MF958299	Hallas et al. 2017
*Kaloplocamus* sp. 3	South Africa	MN968496	MN954202	Toms et al. 2021
*Limaciacockerelli*	USA	KX673492	KX673501	Uribe et al. 2018
*Limaciajanssi*	Mexico	KY622050	KY622048	Uribe et al. 2018
*Limaciamcdonaldi*	USA	KY622051	KY622049	Uribe et al. 2018
*Plocamopherusceylonicus*	Hawaii	KP871650	KP871698	Mahguib and Valdes 2015
*Plocamopherusimperialis*	Australia	JX274103	JX274065	Palomar et al. 2014
*Plocamopherustilesii*	Australia	JX274102	JX274064	Palomar et al. 2014
*Triophacatalinae*	USA	HM162690	HM162600	Pola and Gosliner 2010
*Triophamaculata*	USA	HM162691	HM162601	Pola and Gosliner 2010

## ﻿Results

### ﻿Phylogenetic analyses

**Figure 2.**
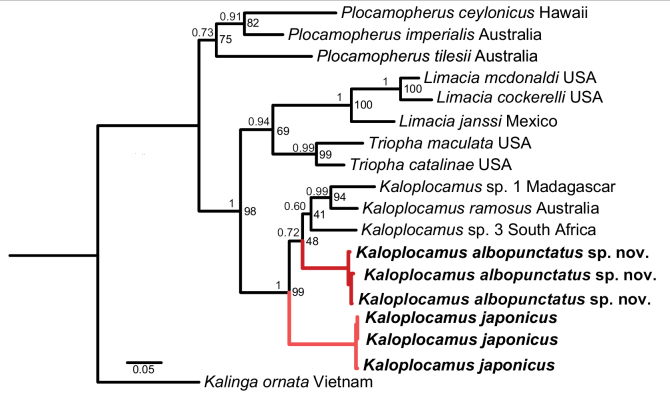
Phylogenetic hypothesis of subfamily Triophinae based on COI and 16S sequences. Sequences obtained in this study in bold. Bootstrap values from ML on the node and posterior probability (PP) values from BI above the branch.

The topology of final concatenated trees from BI and ML analyses were completely congruent, with both species placed clearly within the *Kaloplocamus* clade (Fig. 2). *Kaloplocamusalbopunctatus* sp. nov. is sister to the clade including *K.ramosus* collected in Australia, *K.* sp. 1 from Madagascar, and *K.* sp. 3 from South Africa, while *K.japonicus* is placed in a more basal position that is sister to all the other species of *Kaloplocamus*. The *p*-distances of the COI genes among *K.japonicus* and its congeners ranged from 12.7% to 15.6% (Table 2). In the case of *K.albopunctatus* sp. nov., the *p*-distances within the *Kaloplocamus* clade reached 15.3% when compared with *K.japonicus*, and the minimum distance was 9.6% when compared with *K.ramosus* from Portugal. Notably, we found that the *p*-distances between the sequences of *K.ramosus* from Portugal and from Australia was 9.6%. Moreover, the ASAP analysis also recovered five partitions (Suppl. material 1) with the lowest score (the most supported partition scheme): *K.ramosus* from Australia and Portugal were in different partitions, while *K.ramosus* from Portugal and *Kaloplocamus* sp. 3 from South Africa were clustered together. However, *K.albopunctatus* sp. nov. and *K.japonicus* were supported as distinct species from other *Kaloplocamus* species in all analyses.

**Table 2.** *P*-distance among *Kaloplocamus* species based on COI sequences.

Species label	1	2	3	4	5
1 *K.albopunctatus* sp. nov.					
2 *K.japonicus*	0.153				
3 *K.ramosus* Portugal	0.096	0.127			
4 *K.ramosus* Australia	0.121	0.131	0.096		
5 *K.* sp. 1	0.139	0.156	0.103	0.102	
6 *K.* sp. 3	0.122	0.145	0.074	0.111	0.117

### ﻿Systematics

**Family Polyceridae Alder & Hancock, 1845**

**Genus *Kaloplocamus* Bergh, 1880**

#### ﻿ Kaloplocamus albopunctatus sp. nov.

Taxon classificationAnimaliaNudibranchiaPolyceridae

18CAA66D-5294-548B-81E4-B764C19B3990

https://zoobank.org/C4814283-1992-489A-8BD4-68F82CACFE14

Figs 3 , 4 , 5

Kaloplocamus ramosus : Qi et al. 1986: 63; Baba 1989: 232–234, figs 1–3; Qi et al. 1989: 119, fig. 88; Zhang et al. 2016: 207, fig. 255.

##### Type locality.

China, Shandong: Tianheng Town, 36°33.7′N, 120°58.6′E.

***Holotype***: Alcohol-fixed, original label: “CN, SD, Tianheng, 36°33.7′N, 120°58.6′E, 26 Apr. 2022, J.C. Wei” “MBM287619 ”.

***Paratypes***: Alcohol-fixed, two specimens, original label: “CN, SD, Tianheng, 36°33.7′N, 120°58.6′E, 26 Apr. 2022, J.C. Wei” “LSGB hb266000 0302 to 0303”; alcohol-fixed, five specimens, original label: “CN, SD, Tianheng, 36°33.7′N, 120°58.6′E, 04 May. 2022, J.C. Wei” “LSGB hb266000 0304 to 0308”.

##### Description.

**Figure 3.**
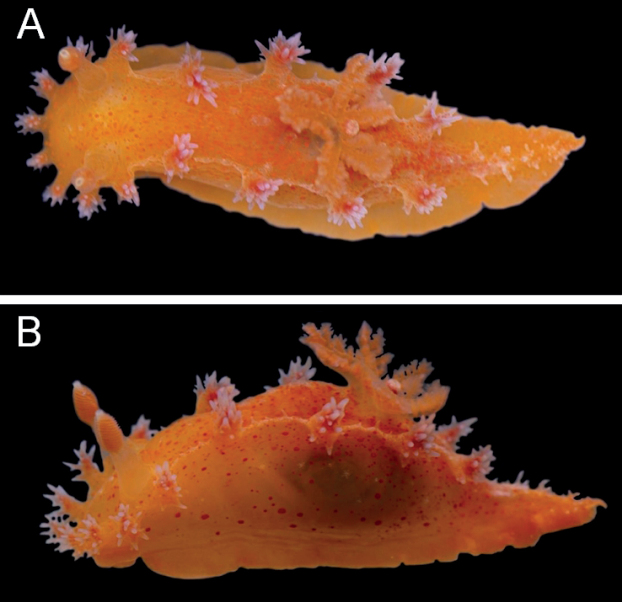
Holotype of *Kaloplocamusalbopunctatus* sp. nov., living specimen, 22 mm in length **A** dorsal view **B** lateral view.

***External morphology*** (Fig. 3) Living animals are up to 25 mm long. The body is elongate, limaciform, bright orange, and with the whole body covered with reddish dots except the foot. These dots are especially dense in the portions around the bases of the lateral appendages and branchial leaves. The lateral portions of the body have scattered white patches composed of dots in series and are moderately transparent so that the digestive gland is visible. The mantle is rather smooth, with very few small, white tubercles on the dorsum. The oral tentacles are flat and wide. The head bears eight velar appendages, which are short, pale orange-yellow, semitranslucent with reddish dots, and the tips are a brighter orange-red color. There are clusters of short, acute ramifications on the velar appendages. The bases of these ramifications are orange-red, while the rest is cream-colored. The dorsum has four pairs of lateral appendages that are larger than the appendages on the head, also with clusters of ramifications. These dorsal appendages share almost the same shape and coloration as the velar ones except that each of the dorsal appendages bears more ramifications. The gill is situated between the second and third pair of appendages. There are five tripinnate branchial leaves and they have the same coloration as the body; the stems of the leaves are translucent and have bright orange dots. The rhinophores have a semitranslucent peduncle with small, dense, orange-yellow dots. The clavus is orange in color, with approximately 16–18 rhinophoral lamellae and with a white tip and a white line on the anterior side. The rhinophoral sheath is short and speckled with orange-yellow dots. The posterior part of the foot is elongate, acutely pointed, and red-orange, with many opaque, white, pointed tubercles that are concentrated centrally.

**Figure 4.**
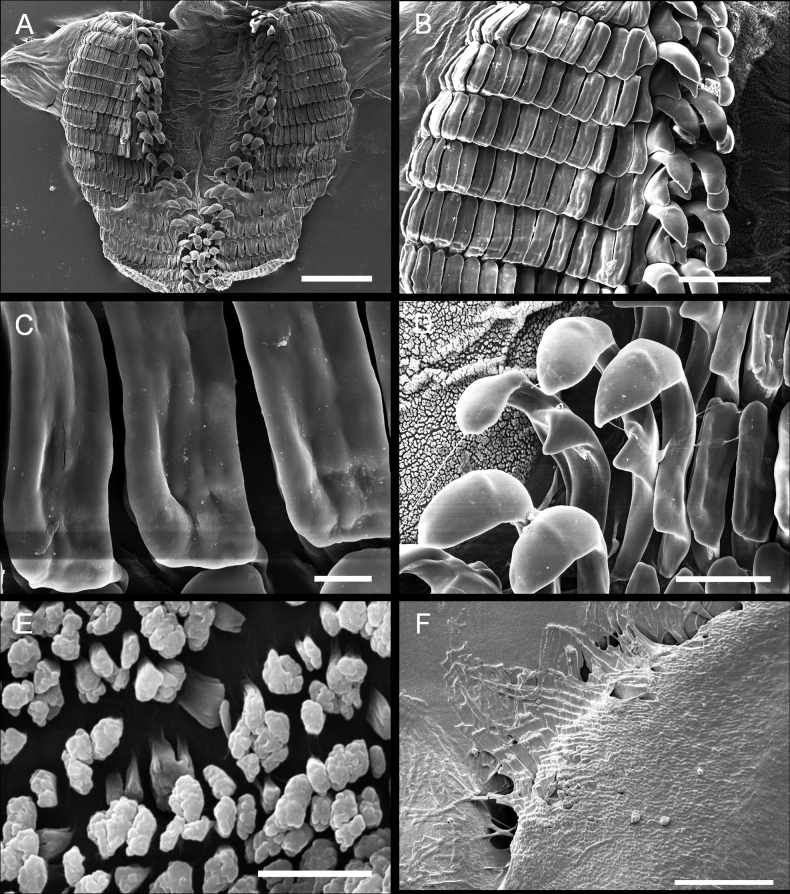
Paratype LSGB hb266000 0305 of *Kaloplocamusalbopunctatus* sp. nov. SEM photographs of the radula and jaws **A** complete radula **B, C** outer marginal teeth **D** inner lateral teeth **E** rachis **F** jaws. Scale bars: 500 μm (**A**); 200 μm (**B**); 100 μm (**D**); 20 μm (**C**, **E**); 100 μm (**F**).

***Radula*** (Fig. 4) The radular formula of the 23 mm specimen examined is 15 × 13.3.0.3.13. The radula is characterized by the presence of two types of well-differentiated teeth (Fig. 4A–D). The three inner lateral teeth share a similar hook-like shape, and a secondary cusp is developed although only the outermost one has a short, less obvious cusp. The hook of the outermost lateral teeth is sharp while on the innermost two teeth, it is comparatively blunt (Fig. 4A, D). The outer marginal teeth are roughly rectangular in shape, with long grooves on the surface (Fig. 4C). The sizes of these teeth are equivalent except for the teeth on the very edge, which are much smaller than the others (Fig. 4B, C). The rachis is granulated and is not transversally divided in rows (Fig. 4E). The jaws have thin, elongate rodlets which are densely packed (Fig. 4F).

**Figure 5.**
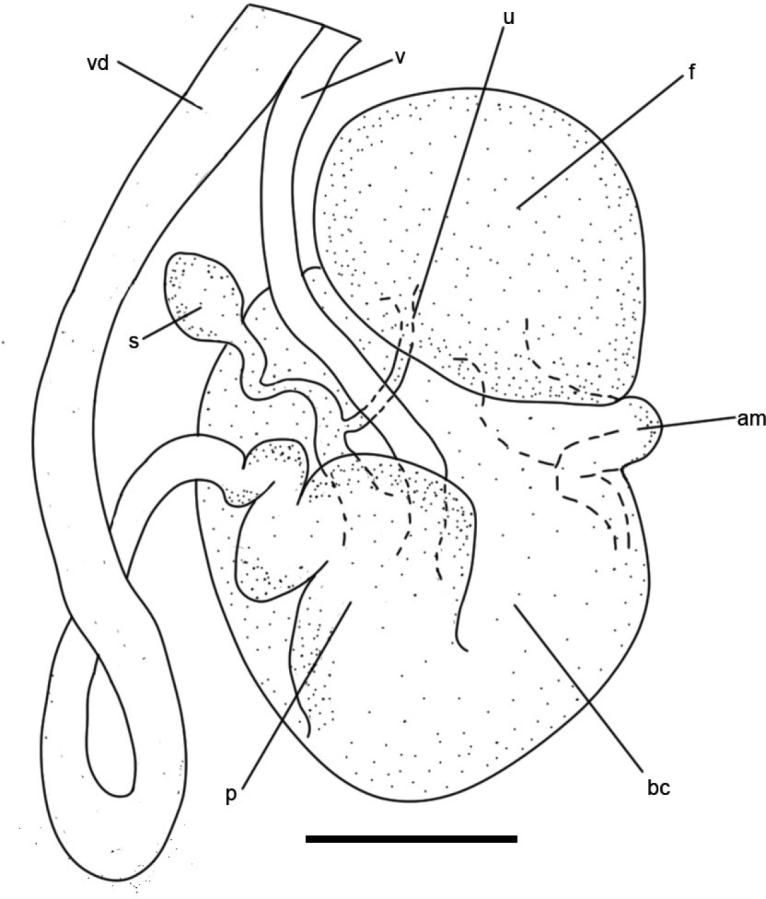
Paratype LSGB hb266000 0305 of *Kaloplocamusalbopunctatus* sp. nov., diagram of the reproductive anatomy. Abbreviations: am – ampulla; bc – bursa copulatrix; f – female gland mass; p – prostate; s – receptaculum seminis; u – uterine duct; v – vagina; vd – vas deferens. Scale bar: 1000 μm.

***Reproductive system*** (Fig. 5) The reproductive system is triaulic. The vas deferens is enlarged in the distal portion and differentiated into an inflated prostate which joins the bursa copulatrix via a thin duct. The vagina is rather narrow, only one-third of the length of the vas deferens, and enters directly to the oval bursa copulatrix. From the bursa copulatrix emerges a short duct that divides into a uterine duct, which connects to the oval female gland mass, and a crooked duct connecting to the receptaculum seminis. The ampulla is short, coiled, and enters into the female gland mass in a relatively distal position. The penis has spines which are elongated and of equal width (Suppl. material 2).

***Egg mass*** (Suppl. material 3). The egg mass of this species is flat, belt-like, coiled, and translucent with white eggs within. It is so similar to the egg mass of *K.japonicus* that DNA was extracted after all the egg masses were photographed and the COI gene was then amplified and sequenced to distinguish the egg masses of these two species.

##### Etymology.

The specific epithet albopunctatus refers to the white patch on the body of this animal. The Chinese common name for this species is “白斑鬈发海牛.”

##### Remarks.

*Kaloplocamusalbopunctatus* sp. nov. shows a bright orange coloration, which is rather common within *Kaloplocamus*. There are four other *Kaloplocamus* species that share a similar color pattern: *K.ramosus*, *K.acutus*, *K.peludo* Vallès & Gosliner, 2006, and *K.maru* Vallès & Gosliner, 2006 (Table 3). Baba (1989) described three forms of *K.ramosus* from Japan: the orange form A shared many similarities with *K.albopunctatus* sp. nov., such as the color of appendages and characters of radula. Baba (1989: 234) described this form as “Sometimes there are a few opaque white spots near the root of the dorso-lateral processes.”, which is a major difference that makes *K.albopunctatus* sp. nov. distinct from *K.ramosus*. In Baba’s drawing of the reproductive system (Baba 1989: fig. 3), the receptaculum seminis (“c” in Baba’s drawing) is not connected to the female gland mass (“fg” in Baba’s drawing). The receptaculum seminis of *K.albopunctatus* sp. nov. is connected to the female gland mass via the uterine duct. However, according to Bergh (1880) and Vallès and Gosliner (2006), it seems to be a common pattern that the receptaculum seminis of *Kaloplocamus* is connected to the bursa copulatrix and the female gland mass. There are many similarities between *Kaloplocamusalbopunctatus* sp. nov. and *K.ramosus*, such as having the same number of ramified appendages and the same color of the body, rhinophores, and branchial leaves, which is why *K.ramosus* had been reported from the Yellow Sea in the past (Qi et al. 1986, 1989; Zhang et al. 2016) despite the type locality of this species being the Mediterranean Sea. However, there are several characters that make *K.albopunctatus* sp. nov. distinct from *K.ramosus*. Firstly, *K.ramosus* is characterized by having small, translucent, orange tubercles on the dorsum and appendages with orange, elongated secondary ramifications, while the dorsum of *K.albopunctatus* sp. nov. is rather smooth and has only short simple ramifications that are cream-colored. Additionally, the vas deferens of *K.albopunctatus* sp. nov. is differentiated into a prostate, but *K.ramosus* does not have a prostate (Vallès and Gosliner 2006). Moreover, the *K.ramosus* reported from the Yellow Sea were described as having “red-orange and white dots on the body, the white dots are usually clustered into a big white patch” (Qi et al. 1986: 63, 1989: 119) or “there are orange and white dots spread on the body” (Zhang et al. 2016: 207), which is exactly the character that *K.albopunctatus* sp. nov. has and *K.ramosus* does not. In consequence, we consider that *K.ramosus* previously documented from the Yellow Sea is a misidentification of *K.albopunctatus* sp. nov.

**Table 3.** Differences between six *Kaloplocamus* species.

Species	Velar appendages	Prostate	Radula	Coloration
*K.ramosus*	8	Absent	17 × 17.6.0.6.17	Orange-red with translucent orange tubercles
*K.japonicus*	8	Present	24 × 17.3.0.3.17	Translucent with pink dots
*K.albopunctatus*	8	Present	15 × 13.3.0.3.13	Bright orange with red and white dots
*K.acutus*	6	Absent	16 × 11.3.0.3.11	Orange-red with white dots
*K.peludo*	6	Present	11 × 8.3.0.3.8	Orange with brown dots
*K.maru*	4	Absent	68 × 7.2.0.2.7	Orange with a white diamond-shaped spot

*Kaloplocamusalbopunctatus* sp. nov. differs from *K.acutus* by the presence of the prostate and in not having bright, carmine-red ramifications and white dots on the entire dorsum. According to Baba (1989), *K.acutus* has two lateral teeth, while *K.albopunctatus* sp. nov. has three lateral teeth. Additionally, *K.acutus* has six velar appendages while *K.albopunctatus* sp. nov. has eight velar appendages on the head. *Kaloplocamuspeludo* has an opaque white, irregularly shaped line and brown dots on the dorsum and bears two types of appendages. Conversely, *K.albopunctatus* sp. nov. does not have these morphological characters and the appendages are uniform rather than differentiated into two types. Finally, *K.albopunctatus* sp. nov. can be distinguished from *K.maru* by the vas deferens that is differentiated into a prostate and the absence of the white diamond-shaped spot on the dorsum. Another feature that distinguishes *K.albopunctatus* sp. nov. from *K.maru* is that *K.maru* has a long ribbon of teeth with 68 rows of teeth on it, which clearly differs from the ribbon of *K.albopunctatus* sp. nov. that only has 24 rows of teeth.

#### ﻿ Kaloplocamus japonicus

Taxon classificationAnimaliaNudibranchiaPolyceridae

Bergh, 1880

EBB6D274-82C0-5A93-AC0D-21824CB7EE65

Figs 6 , 7 , 8 , 9

Euplocamus japonicus : Bergh, 1880, 32, pl. XIII, fig. 17, pl. XIV, figs 3–10.

##### Type locality.

Japan.

##### Material examined.

Alcohol-fixed, two specimens, original label: “CN, SD, Tianheng, 36°33.7′N, 120°58.6′E, 04 May. 2022, J.C. Wei” “LSGB hb266000 0201 to 0202”; alcohol-fixed, three specimens, original label: “CN, SD, Tianheng, 36°33.7′N, 120°58.6′E, 04 May. 2022, J.C. Wei” “LSGB hb266000 0203 to 0205”.

##### Description.

**Figure 6.**
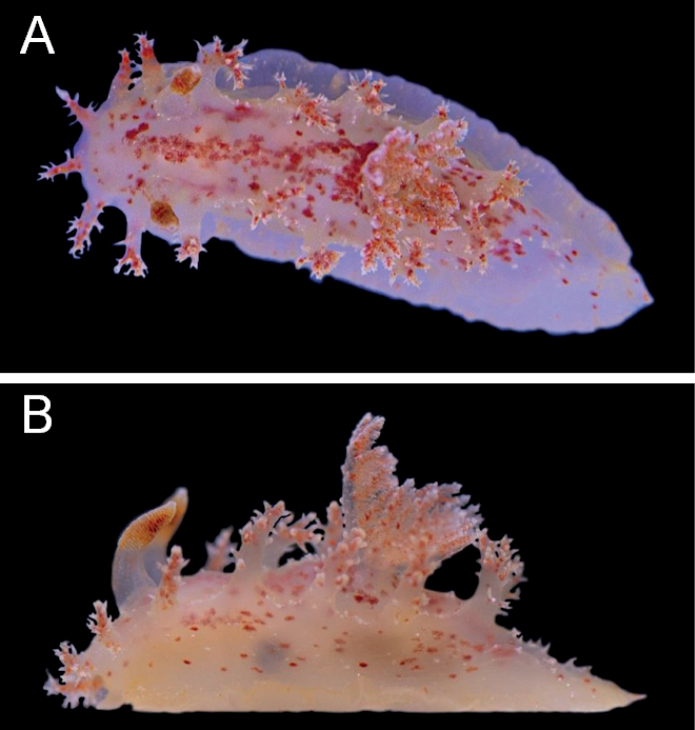
*Kaloplocamusjaponicus*, living specimen, 18 mm in length. **A** dorsal view **B** lateral view.

***External morphology*** (Fig. 6) Living animals are up to 21 mm in length. The body is elongate, limaciform, and translucent white with pink dots. The entire dorsum is covered in small, opaque, white tubercles. The oral tentacles are flat and thin. The head bears eight velar appendages, approximately half of the length of the rhinophores. These appendages are also translucent white and have elongate, sharp, secondary ramifications. There are four pairs of lateral appendages on the dorsum that are approximately twice as long as the ones on the veil, and they have thicker ramifications. All the appendages share the same coloration as the body. The branchial leaves are situated between the second and third pair of appendages. There are five tripinnate branchial leaves which have the same coloration as the body; the stalks of the branchial leaves have small, dense, white protuberances. The rhinophores have a semitranslucent stalk and an orange-red lamellate clavus with approximately 26–28 rhinophoral lamellae; the clavus has a white tip and a white line on the anterior side. The rhinophoral sheath is short and has small, opaque, white tubercles around its margin.

**Figure 7.**
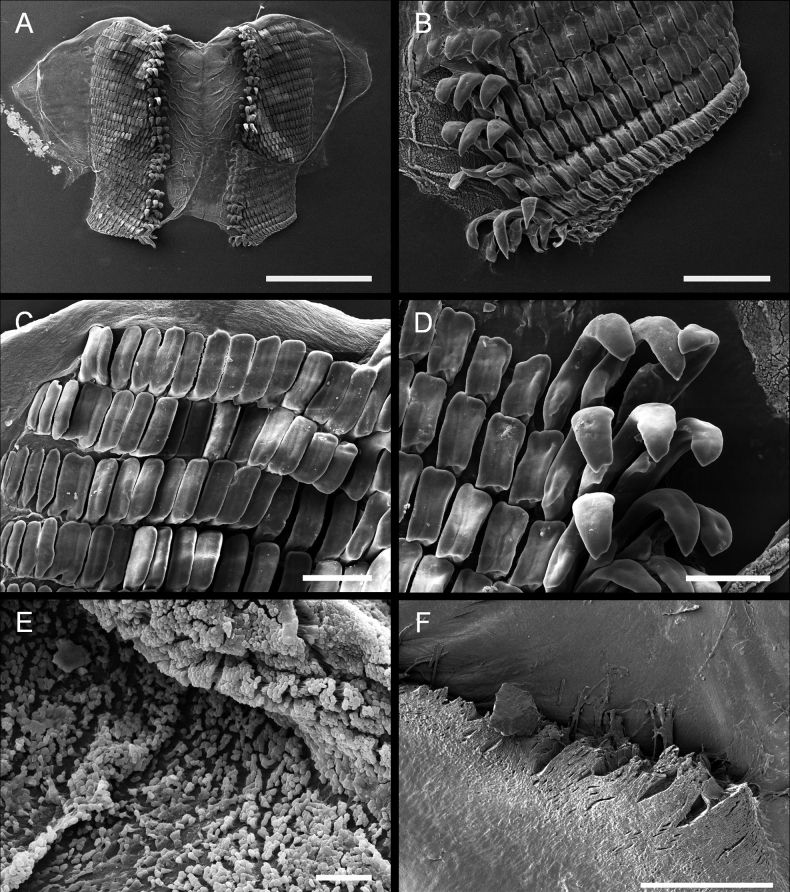
Specimen LSGB hb266000 0203 of *Kaloplocamusjaponicus*, SEM photographs of the radula and jaws **A** whole radula **B, C** outer lateral teeth **D** inner lateral teeth **E** rachis **F** jaws. Scale bars: 1000 μm (**A**); 200 μm (**B**); 100 μm (**C, D**); 20 μm (**E**); 200 μm (**F**).

***Radula*** (Fig. 7) The radula formula of a 20 mm specimen is 24 × 17.3.0.3.17, and this species is characterized by two different types of teeth (Fig. 7A–D). There are three inner lateral teeth per side; the innermost two teeth are similar in shape and have a sharp, hook-like portion at the apex, and the tip is not bifurcated, while the third one has a blunt bifurcation on the tip (Fig. 7D). All the inner lateral teeth have a secondary cusp, but this structure on the third tooth is not as pronounced as that of the innermost two. The outer marginal teeth are flat and roughly rectangular, and the size of these teeth decreases along the row (Fig. 7C). The rachis is granulated and does not transversally divide into rachidian plate rows (Fig. 7E). The jaws have thin, elongate, laminar rodlets which are densely packed (Fig. 7F).

**Figure 8.**
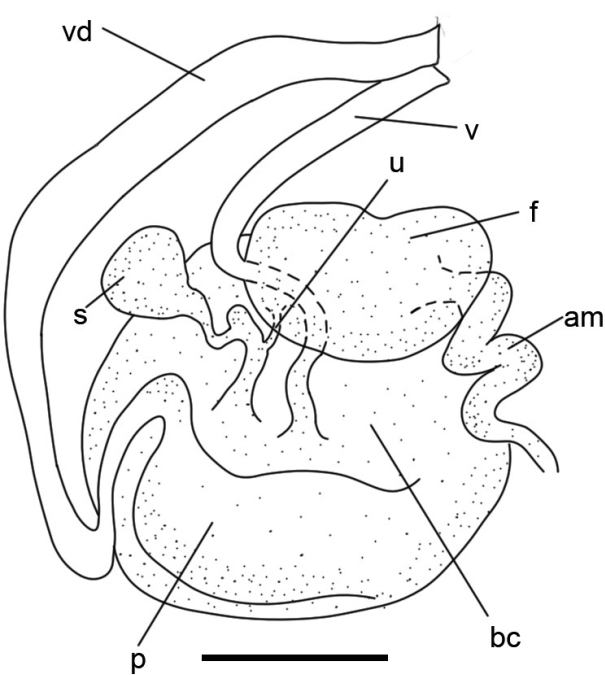
Specimen LSGB hb266000 0203 of *Kaloplocamusjaponicus*, diagram of the reproductive anatomy. Abbreviations: am – ampulla; bc – bursa copulatrix; f – female gland mass; p – prostate; s – receptaculum seminis; u – uterine duct; v – vagina; vd – vas deferens. Scale bar: 1000 μm.

***Reproductive system*** (Fig. 8) The reproductive system is triaulic (Fig. 8). The vas deferens is differentiated into an oblong-oval prostate in its distal portion, which is connected to the oval bursa copulatrix. The vagina is thin and approximately half of the length of the vas deferens, entering directly into the bursa copulatrix. From the bursa copulatrix protrudes a short, thick duct with an ampoule-like extension; the female gland mass is connected to that duct via the uterine duct, and on the other side of it, a crooked duct exits that connects to the oval receptaculum seminis. The ampulla is long, coiled, and enters into the female gland mass in a relatively distal position. The penis has spines and is slightly inflated in the distal position (Suppl. material 4).

***Egg mass*** (Suppl. material 5). The egg mass of this species is flat, belt-like, coiled, and translucent with white eggs within.

##### Remarks.

This species is characterized by several features that are very different from those of the other known species of *Kaloplocamus*. The most apparent one is its translucent white-pink body coloration, which has never been reported before. Additionally, the way its bursa copulatrix connects to the receptaculum seminis and female gland mass is also different. Most *Kaloplocamus* species have a Y-shaped structure composed of three ducts of approximately the same width which link these organs, while *K.japonicus* has an ampoule-like extension on the duct connected to the bursa copulatrix. The extension is approximately twice as wide as the uterine duct and protrudes from the join (Fig. 8). This structure was also described by Bergh (1880) and is the main reason we consider our specimens to be *K.japonicus*. Although Bergh (1880) described *K.japonicus* as having six appendages on head, it is likely that the last pair of velar appendages were misidentified as dorsal appendages considering that he noted *K.japonicus* has five pairs of appendages on the dorsum. Eliot (1913) also reported two specimens from Japan, but the coloration of his specimens was not mentioned because they were fixed in alcohol when he examined them. The structure of reproductive system was also absent, and it is impossible to know if the specimens were *K.japonicus*. The color forms B and C described by Baba (1989) resemble *K.japonicus* in coloration; however, the reproductive system structures of these forms remain unknown and more evidence is needed to determine whether they are *K.japonicus* or other undescribed *Kaloplocamus* species.

**Figure 9.**
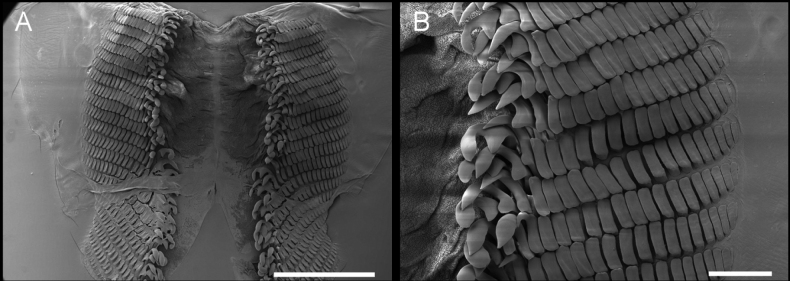
Specimen LSGB hb266000 0204 of *Kaloplocamusjaponicus*, SEM photographs of the radula **A** whole radula **B** outer lateral teeth. Scale bars: 1000 μm (**A**); 200 μm (**B**).

Interestingly, there seems to be an aberration on the radula of the specimen examined (Fig. 7C). The two outer marginal teeth on each row of the left side merges into one, making that side have only 16 outer lateral teeth, while on the right side there are 17 outer marginal teeth without a merged tooth. The merged tooth is approximately twice the width of the largest outer lateral teeth and is erupted from two independent roots. These merged teeth are found in every row of the left side of the radula and this has neither been reported in other species of *Kaloplocamus* nor found in examined *K.japonicus* specimens (Fig. 9A, B).

## ﻿Discussion

### ﻿Bioluminescence

The genus *Kaloplocamus* is known for the ability to bioluminesce; however, only *Kaloplocamusramosus* is reported to emit light when disturbed (Vallès and Gosliner 2006), and it is supposed that the light could serve as a distraction when encountering a predator. We maintained five *K.albopunctatus* sp. nov. and three *K.japonicus* in a seawater tank from 5 May to 3 June 2022, and during this period these animals were regularly provoked by a pair of iron tweezers with the light of the laboratory off, simulating a potential predator, and no bioluminescent phenomenon was observed. However, it is still unwise to affirm that they are incapable of producing light according to Vallès and Gosliner (2006), because even bioluminescent species stopped emitting light when under constant physical disturbance or stressed. Further in-the-wild observations of these species are required to determine their capability of bioluminescence.

### ﻿*Kaloplocamusramosus* species complex

Despite the fact that Vallès and Gosliner (2006) synonymized four *Kaloplocamus* species with *K.ramosus*, they revised *Kaloplocamus* only based on morphological characters. Therefore, a thorough study of comprehensive morphological and molecular data of all the putative “*K.ramosus*” species was recommended. According to current results, it seems premature to make these synonymizations because there appears to be a species complex surrounding *K.ramosus* with at least three potential species (Suppl. material 1). The *K.ramosus* reported in the Yellow Sea is verified as a new species, *K.albopunctatus* sp. nov., based on our morphological examinations and molecular analyses. Our ASAP analysis also indicates that the species called *K.ramosus* from both Australia and Portugal are likely separate species, as their genetic divergence is beyond that of the intraspecies level; whether they are true *K.ramosus* or other *Kaloplocamus* species needs further study. However, the synonymized names are poorly described and the type specimens are not properly conserved, raising even more difficulties to resurrect these names.

### ﻿New phylogenetic relationship of *Kaloplocamus* and *Plocamopherus*

Vallès and Gosliner (2006) stated that a phylogenetic analysis of Triophinae may shed light on the evolution of bioluminescence within the dorids because if *Kaloplocamus* and *Plocamopherus* were sister groups, it could be assumed that this ability had evolved just once in Triophinae dorids. Palomar et al. (2014) conducted the first molecular phylogenetic work on polycerids based on the mitochondrial COI and 16S genes with a polytomy including the genera *Kaloplocamus*, *Plocamopherus*, and *Triopha*. Hallas et al. (2017) used a multiple sequence data set (COI, 16S, 18S, 28S) to carry out phylogenetic analyses of the Nudibranchia, concluding that *Kaloplocamus* was clustered with *Plocamopherus* in all analyses, and that this clade would be sister to *Triopha*, *Limacia*, or the clade including *Triopha* and *Limacia* depending on different multiple sequence alignment approaches. The same multiple sequence data set (COI, 16S, 18S, 28S) was applied by Korshunova et al. (2020) for phylogenetic analyses of the Nudibranchia, and *Kaloplocamus* was again recovered as sister group to *Plocamopherus*, then sister to the clade including *Triopha* and *Limacia*. It is widely recognized that wider taxon sampling will improve the phylogenetic results, and even well-established clades could be refined (Dunn et al. 2008; Tamashiro et al. 2019; Uribe et al. 2019; Xu et al. 2022). By adding more taxa (Table 1), we conducted phylogenetic analyses based on a data set consisting of concatenated COI and 16S genes of all the available sequences including 13 Triophinae species. We recovered the genus *Kaloplocamus* as the sister to the clade that includes *Triopha* and *Limacia* with high support (99% PP and 98% BS), and the genus *Plocamopherus* was placed on the most basal position of Triophinae (Fig. 2). Our result indicates that the bioluminescence of Triophinae dorids might have experienced two independent origins and is potentially supported by the fundamentally different light-emitting systems in *Kaloplocamus* and *Plocamopherus*. In *K.ramosus*, the light emission takes place within the cells (intracellular luminescence) while the light of *Plocamopherus* species was emitted out of the cells (extracellular luminescence) via the luminous chemicals produced by a sort of special cell (Vallès and Gosliner 2006). If the ability of bioluminescence evolved only once, it would be difficult to explain why two closely related taxa emit light in such disparate ways.

**Figure 10.**
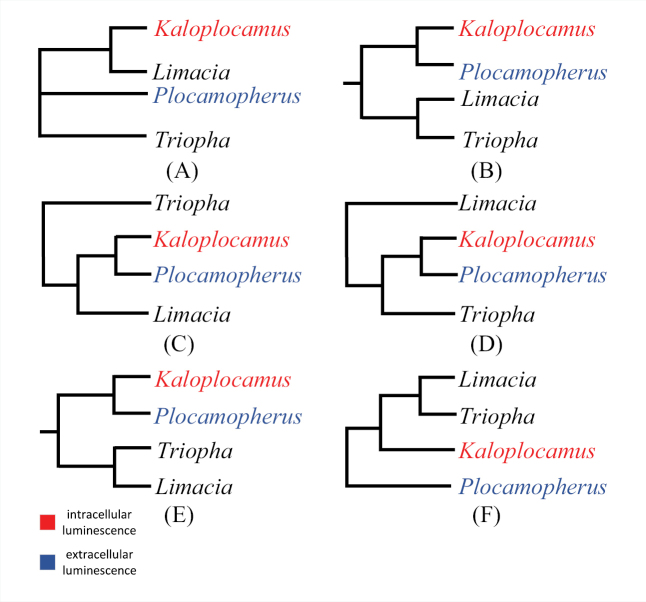
Phylogenetic estimates of bioluminescent genera and their sister groups **A**Palomar et al. (2014)**B**Hallas et al. (2017), sequences aligned by MAFFT **C**Hallas et al. (2017), sequences aligned by Gblocks **D**Hallas et al. (2017), sequences aligned by MAFFT and then modified manually **E**Korshunova et al. (2020)**F** this study, sequences aligned by MAFFT.

## ﻿Conclusions

*Kaloplocamusalbopunctatus* sp. nov. is externally different from previously reported congeners by its coloration patterns and the characters of their appendages. *Kaloplocamusjaponicus* is revised and removed from synonymy with *K.ramosus* based on the characters of reproductive system. The internal anatomy examination, SEM photographs, and molecular analyses also provide further reliable evidence that delimits *Kaloplocamusjaponicus* and *Kaloplocamusalbopunctatus* sp. nov. as valid species within the *Kaloplocamus* lineage.

The relationships among the taxa within Triophinae have also been reconstructed based on a finer taxon sampling, with *Plocamopherus* placed at the base of Triophinae while *Kaloplocamus* is considered to be sister to *Triopha* and *Limacia*. Our result brings new insights to the evolution of the bioluminescence of polycerids, indicating that this ability may had evolved twice, independently, and potentially explains the reason the light emitting systems of *Kaloplocamus* and *Plocamopherus* are intrinsically different.

The formerly reported *K.ramosus* in the Yellow Sea is found to be a misidentification of *K.albopunctatus* sp. nov., but there are still unresolved problems within the *K.ramosus* complex. The ASAP species delimitation method has recovered at least three possible species, with *K.albopunctatus* sp. nov. being one of them. To solve these problems thoroughly, an integrated analysis combining morphology and molecular data of the specimens from all the reported locations of *K.ramosus* complex is needed.

## Supplementary Material

XML Treatment for Kaloplocamus albopunctatus

XML Treatment for Kaloplocamus japonicus

## ﻿Additional information

### Author ORCIDs

Jingcheng Wei https://orcid.org/0000-0003-0619-163X

Lingfeng Kong https://orcid.org/0000-0001-5263-1697

### Data availability

All of the data that support the findings of this study are available in the main text or Supplementary Information.

## Supplementary materials

Supplementary material 1 ASAP analysis for the *Kaloplocamusramosus* complex based on COI sequences Data type image Explanation note ASAP analysis for the *Kaloplocamusramosus* complex based on COI sequences, five partitions were recovered with the lowest score (the most supported partition scheme). https://binary.pensoft.net/file/869438This dataset is made available under the Open Database License (http://opendatacommons.org/licenses/odbl/1.0/). The Open Database License (ODbL) is a license agreement intended to allow users to freely share, modify, and use this Dataset while maintaining this same freedom for others, provided that the original source and author(s) are credited.Jingcheng Wei, Lingfeng Kong

Supplementary material 2 Penis of of *Kaloplocamusalbopunctatus* sp. nov. Data type image Explanation note The penis of *Kaloplocamusalbopunctatus* sp. nov. Scale bar: 100 μm. https://binary.pensoft.net/file/869439This dataset is made available under the Open Database License (http://opendatacommons.org/licenses/odbl/1.0/). The Open Database License (ODbL) is a license agreement intended to allow users to freely share, modify, and use this Dataset while maintaining this same freedom for others, provided that the original source and author(s) are credited.Jingcheng Wei, Lingfeng Kong

Supplementary material 3 Egg mass of *Kaloplocamusalbopunctatus* sp. nov. Data type image https://binary.pensoft.net/file/869440This dataset is made available under the Open Database License (http://opendatacommons.org/licenses/odbl/1.0/). The Open Database License (ODbL) is a license agreement intended to allow users to freely share, modify, and use this Dataset while maintaining this same freedom for others, provided that the original source and author(s) are credited.Jingcheng Wei, Lingfeng Kong

Supplementary material 4 The penis of *Kaloplocamusjaponicus* Data type image Explanation note The penis of *Kaloplocamusjaponicus*. Scale bar: 100 μm. https://binary.pensoft.net/file/869441This dataset is made available under the Open Database License (http://opendatacommons.org/licenses/odbl/1.0/). The Open Database License (ODbL) is a license agreement intended to allow users to freely share, modify, and use this Dataset while maintaining this same freedom for others, provided that the original source and author(s) are credited.Jingcheng Wei, Lingfeng Kong

Supplementary material 5 Egg mass of *Kaloplocamusjaponicus* Data type image https://binary.pensoft.net/file/869442This dataset is made available under the Open Database License (http://opendatacommons.org/licenses/odbl/1.0/). The Open Database License (ODbL) is a license agreement intended to allow users to freely share, modify, and use this Dataset while maintaining this same freedom for others, provided that the original source and author(s) are credited.Jingcheng Wei, Lingfeng Kong

Supplementary material 6 Information of specimens Data type table (excel file) Explanation note Registration numbers of specimens, and the information of these specimens. https://binary.pensoft.net/file/869443This dataset is made available under the Open Database License (http://opendatacommons.org/licenses/odbl/1.0/). The Open Database License (ODbL) is a license agreement intended to allow users to freely share, modify, and use this Dataset while maintaining this same freedom for others, provided that the original source and author(s) are credited.Jingcheng Wei, Lingfeng Kong
