# Supplementary material for: ﻿Taxonomic review of Kaloplocamus from the Yellow Sea, China with the description of a new species (Nudibranchia, Doridina, Polyceridae)
Source: Zookeys. 2023 Jun 27;1168:107–29. doi: 10.3897/zookeys.1168.101248 (PMC10320717; doi:10.3897/zookeys.1168.101248)
Supplement: Supplementary material 1 — ASAP analysis for the Kaloplocamusramosus complex based on COI sequences [file zookeys-1168-107_article-101248__-s001.pdf]

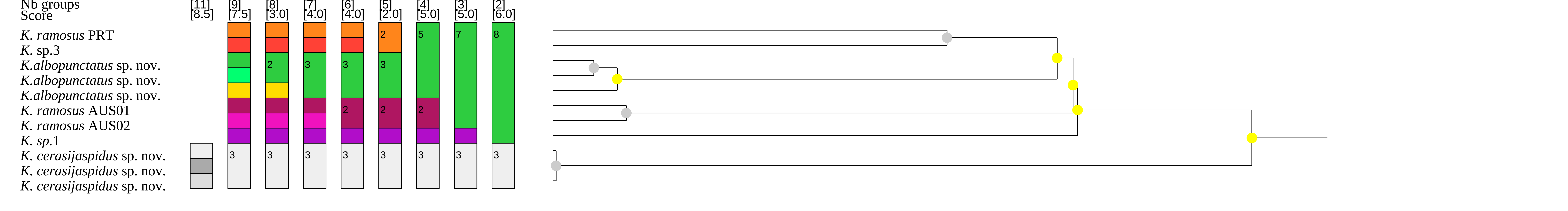
Nb groups
Score
[11] [8.5]
[9] [7.5]
[8] [3.0]
[7] [4.0]
[6] [4.0]
[5] [2.0]
[4] [5.0]
[3] [5.0]
[2] [6.0]
K. ramosus PRT
K. sp.3
K.albopunctatus sp. nov.
K.albopunctatus sp. nov.
K.albopunctatus sp. nov.
K. ramosus AUS01
K. ramosus AUS02
K. sp.1
K. cerasijaspidus sp. nov.
K. cerasijaspidus sp. nov.
K. cerasijaspidus sp. nov.
