# Supplementary figures and images for: ﻿Taxonomic review of Kaloplocamus from the Yellow Sea, China with the description of a new species (Nudibranchia, Doridina, Polyceridae)
Source: Zookeys. 2023 Jun 27;1168:107–29. doi: 10.3897/zookeys.1168.101248 (PMC10320717; doi:10.3897/zookeys.1168.101248)

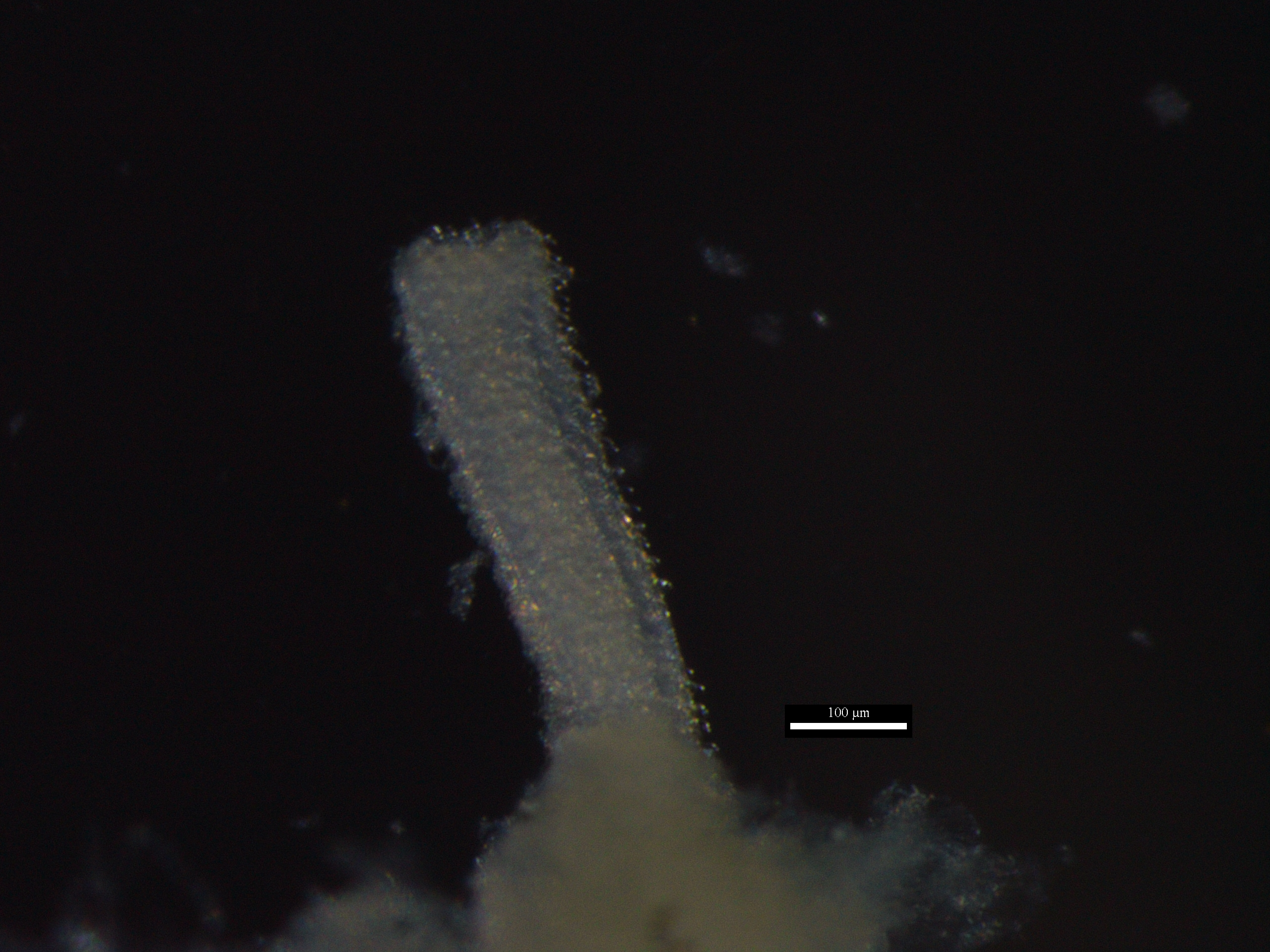

Supplement: Supplementary material 2 — Penis of of Kaloplocamusalbopunctatus sp. nov. [file zookeys-1168-107_article-101248__-s002.jpg]

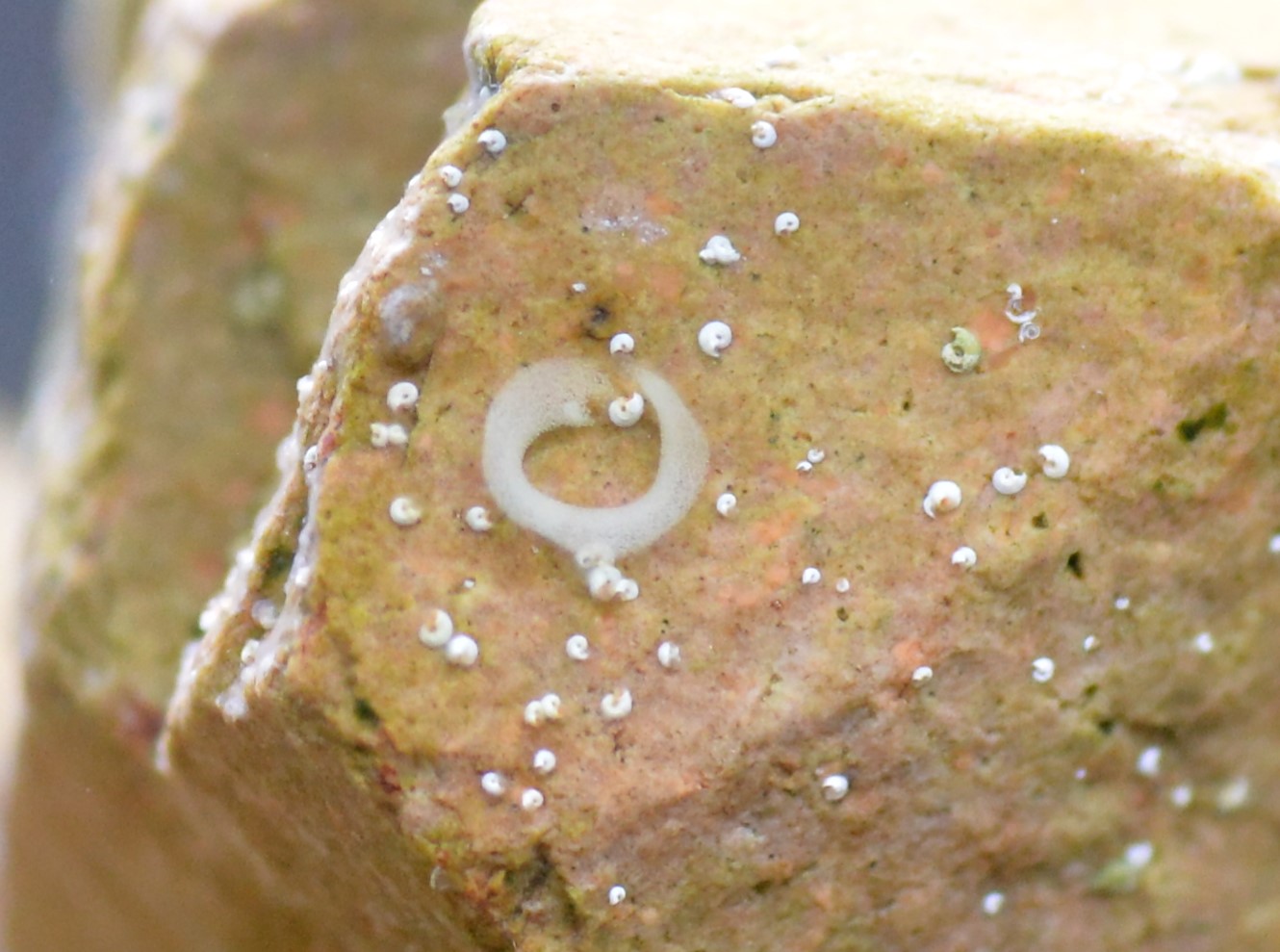

Supplement: Supplementary material 3 — Egg mass of Kaloplocamusalbopunctatus sp. nov. [file zookeys-1168-107_article-101248__-s003.jpg]

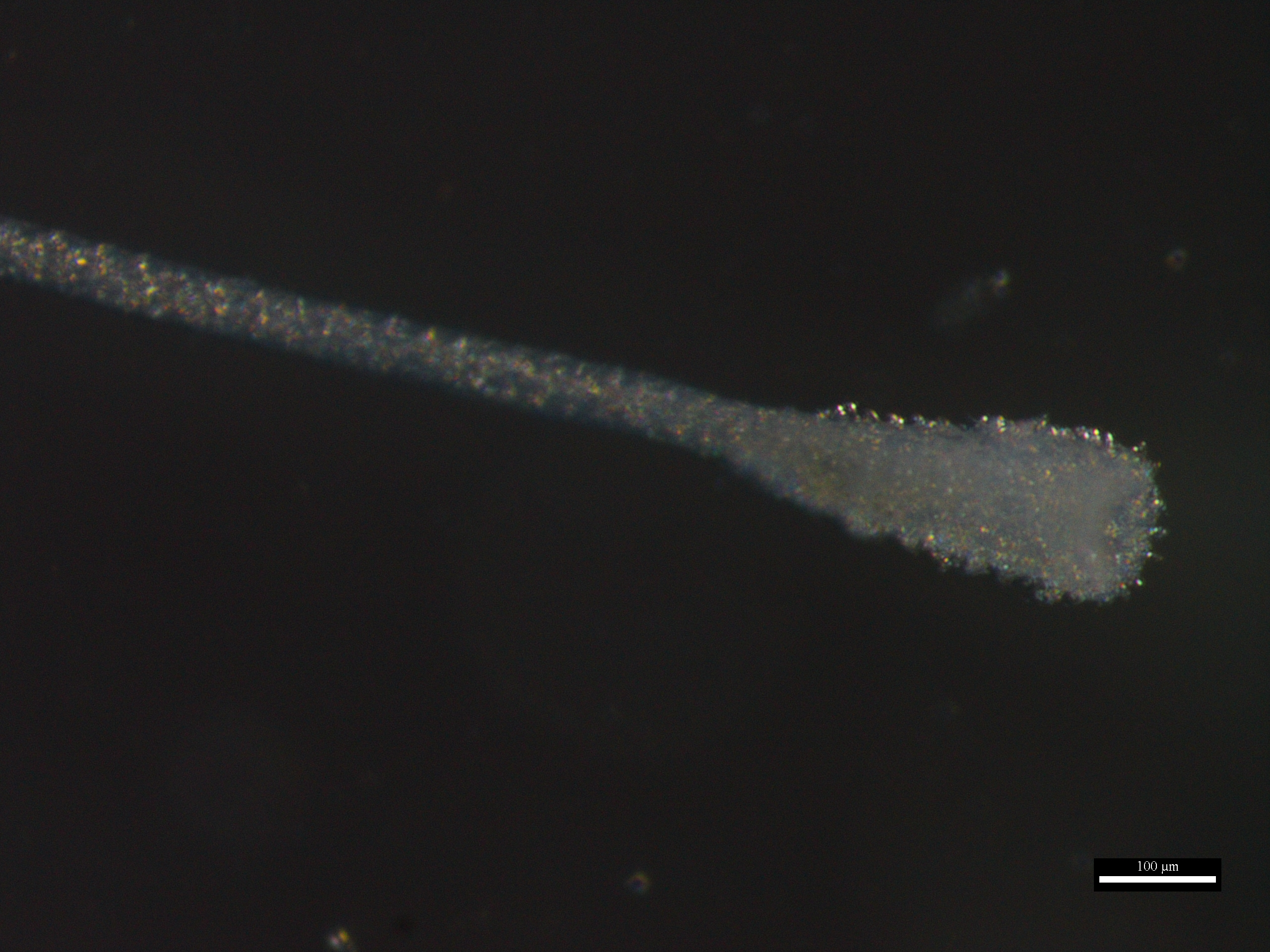

Supplement: Supplementary material 4 — The penis of Kaloplocamusjaponicus [file zookeys-1168-107_article-101248__-s004.jpg]

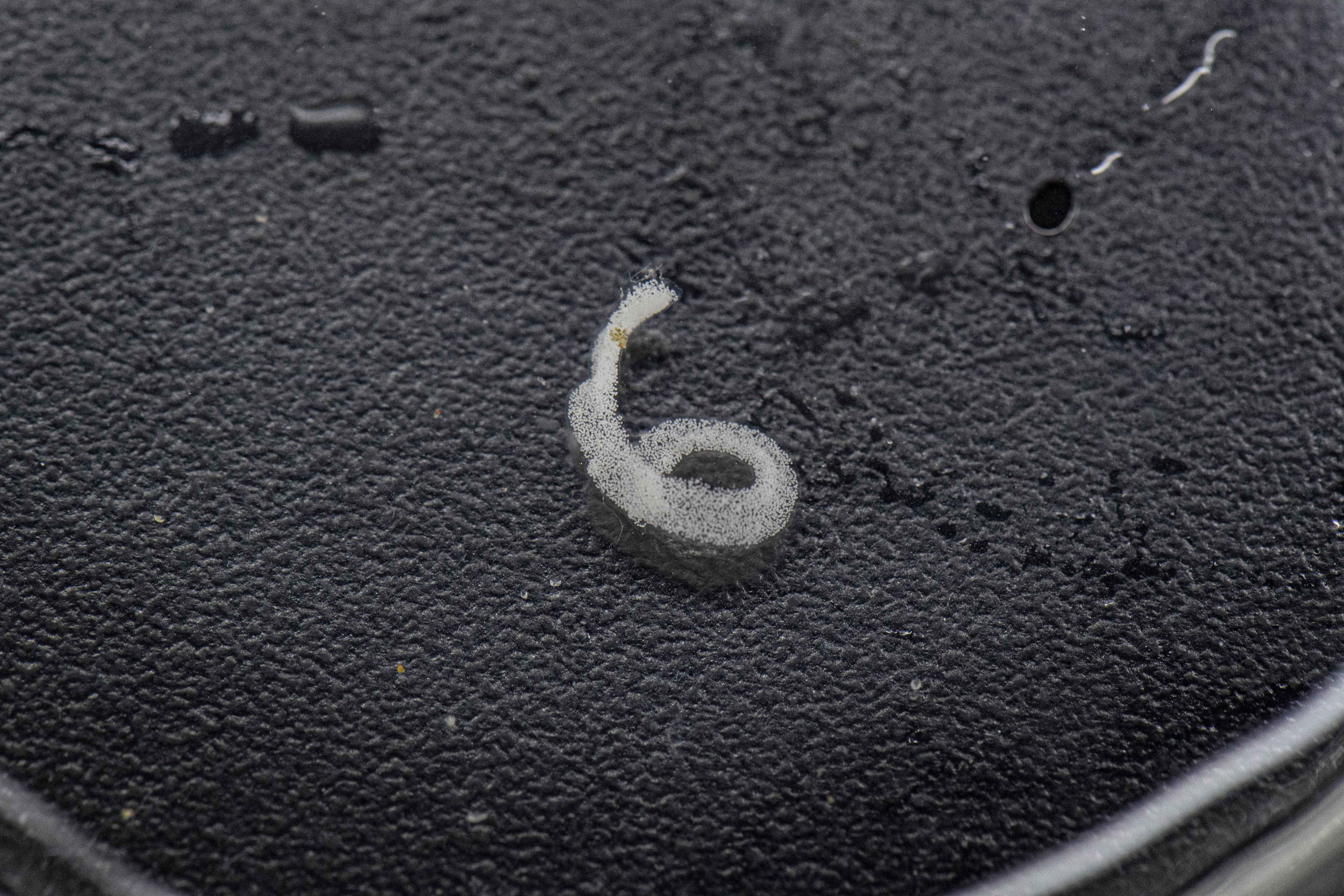

Supplement: Supplementary material 5 — Egg mass of Kaloplocamusjaponicus [file zookeys-1168-107_article-101248__-s005.jpg]
